# Treatment of Insanity

**Published:** 1899-06

**Authors:** 


### TREATMENT OF INSANITY.

Dr. F. W. Langdon, of Cincinnati, in an excellent article on the Treatment of Insanity, refers to the preventive measures to be taken in childhood as follows:

The neurotic child should not be made a man or woman before its time; should not be taken to funerals, theatres, religious revivals and other emotional exhibitions; should not be put in competition with stronger children at school. How often is the opposite course pursued by parents because the child is "bright," which in most cases means "weak," *i. e.*, easily impressed. If association of a child with a hysterical parent is to be condemned, how much more important that it be not brought into intimate association with insane relations! It would be an advantage to many of these neurotic "border-line" children if they could be kept from school until such time as nature has developed their neurons more fully and proper inhibitory connections are made. But, protests the parent, my child must have an education—must hold a position in "society."

Such parents should be warned that an imperfect fruit may be gathered at the expense of loss of the tree. It is our duty to advise parents not to "push" these children at their studies, to take off the pressure and allow the nerve elements to attain the best proportions possible to their nature. A few years spent in the sunshine of the fields and woods, with careful attention to general hygiene and nutrition, are worth more than all the veneer of civilisation to these backward plants."

He summarises the treatment of the insanities as follows :

1. A great majority of first attacks are amenable to home treatment if proper facilites for isolation, nursing, hygiene and medical attention are procurable.

2. Treat the patient not the insanity.

3. Treatment should consist of : (*a*) Causal treatment, removal of excitants and depressants; (*b*) Somatic treatment, eliminative and nutritional; (*c*) Symptomatic treatment, to allay restlessness, to promote sleep, to secure development in adolescence, to favor involution in the climacteric; (*d*) Institutional treatment is advisable in paranoia with dangerous delusions, in recurrent mania with violence, in paresis, in the terminal dementias following other insanities, and in all cases where proper isolation and nursing cannot be afforded, or where the associations are such (children, etc.) as to make the patient a menace to the psychic health of others.—*Cincinnati Lancet Clinic.*

